# A Narrative Review on Adipose Tissue and Overtraining: Shedding Light on the Interplay among Adipokines, Exercise and Overtraining

**DOI:** 10.3390/ijms25074089

**Published:** 2024-04-06

**Authors:** Marta Mallardo, Aurora Daniele, Giuseppe Musumeci, Ersilia Nigro

**Affiliations:** 1 Department of Molecular and Biotechnological Medicine, University of Naples “Federico II”, 80131 Naples, Italy; mallardo@ceinge.unina.it; 2 CEINGE-Biotechnologies Advances S.c.a r.l., Via G. Salvatore 486, 80145 Naples, Italy; ersilia.nigro@unicampania.it; 3 Department of Biomedical and Biotechnological Sciences, Anatomy, Histology and Movement Sciences Section, School of Medicine, University of Catania, Via S. Sofia 87, 95123 Catania, Italy; 4 Research Center on Motor Activities (CRAM), University of Catania, 95123 Catania, Italy; 5 Department of Pharmaceutical, Biological, Environmental Sciences and Technologies, University of Campania “Luigi Vanvitelli”, Via G. Vivaldi 42, 81100 Caserta, Italy

**Keywords:** physical activity, lifestyle, exercise, athletes, adipose tissue, adipokines, overtraining syndrome

## Abstract

Lifestyle factors, particularly physical inactivity, are closely linked to the onset of numerous metabolic diseases. Adipose tissue (AT) has been extensively studied for various metabolic diseases such as obesity, type 2 diabetes, and immune system dysregulation due to its role in energy metabolism and regulation of inflammation. Physical activity is increasingly recognized as a powerful non-pharmacological tool for the treatment of various disorders, as it helps to improve metabolic, immune, and inflammatory functions. However, chronic excessive training has been associated with increased inflammatory markers and oxidative stress, so much so that excessive training overload, combined with inadequate recovery, can lead to the development of overtraining syndrome (OTS). OTS negatively impacts an athlete’s performance capabilities and significantly affects both physical health and mental well-being. However, diagnosing OTS remains challenging as the contributing factors, signs/symptoms, and underlying maladaptive mechanisms are individualized, sport-specific, and unclear. Therefore, identifying potential biomarkers that could assist in preventing and/or diagnosing OTS is an important objective. In this review, we focus on the possibility that the endocrine functions of AT may have significant implications in the etiopathogenesis of OTS. During physical exercise, AT responds dynamically, undergoing remodeling of endocrine functions that influence the production of adipokines involved in regulating major energy and inflammatory processes. In this scenario, we will discuss exercise about its effects on AT activity and metabolism and its relevance to the prevention and/or development of OTS. Furthermore, we will highlight adipokines as potential markers for diagnosing OTS.

## 1. Introduction

**Figure 1:**
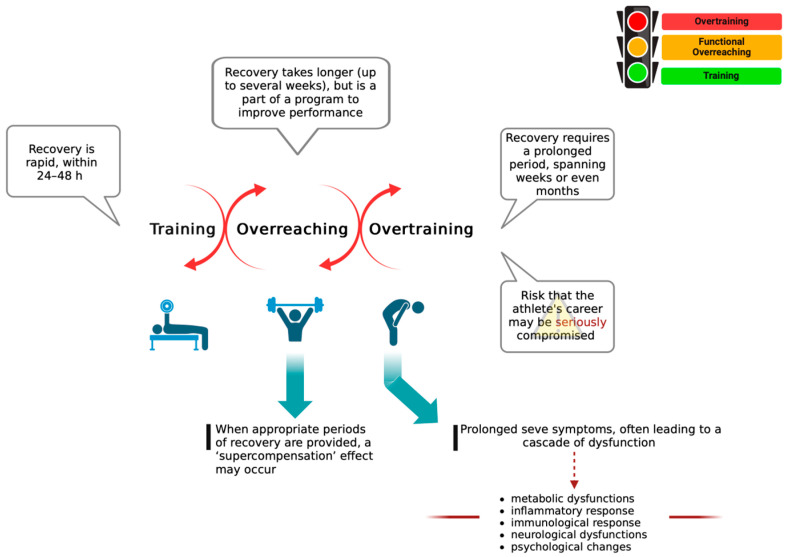
The progression from intensified training to OR and/or OT is commonly regarded as a continuous spectrum. The figure was created using BioRender.com.

In the sports sector, training intensification is a commonly adopted approach to improve athlete performance [1]. However, an adequate balance between training and recovery is essential for athletes to achieve continued high-level performance. Consequently, without sufficient muscle recovery and rest, athletes might experience acute feelings of fatigue and decline in performance. In this context, the athlete can move from adequate training to overreaching (OR) and, ultimately, to overtraining syndrome (OTS) [2]. Prevalence and incidence data for OTS are still lacking due to the diagnostic challenges it presents. It is estimated that the lifetime prevalence of OTS is around 30% among non-elite endurance athletes and 60% among elite athletes [2,3]. Another problem is that it is difficult to distinguish between OR and the early phases of overtraining (OT). Indeed, many consider the transition from optimal training to OR and/or OT as a continuum (see Figure 1).

In addition to decreased performance, many physiological and psychological changes occur in the presence of OTS that can have a negative impact on various systems, including the neuroendocrine, cardiovascular and musculoskeletal systems [4]. Despite numerous theories suggesting the causes of OTS, including glycogen depletion, central fatigue, and glutamine depletion, none of these proposed mechanisms fully account for the development of OTS [5]. Overtrained athletes experienced a wide range of symptoms, including but not limited to increased susceptibility to injury, fatigue, sleep disruption, weight loss, muscle tenderness, weakness, depression, anxiety, difficulty concentrating, and altered eating [2]. In addition, alterations in the immunological and/or inflammatory status, indicated by changes in various indices of immune system functionality and in numerous biochemical parameters, have been identified as common manifestations of OTS [6,7,8]. Physical exercise and OTS determine a series of physiological adaptations in many organs and tissues, including adipose tissue (AT) [7]. It is known, in fact, that following physical exercise, AT undergoes remodeling, activating lipolysis and reducing the size and/or number of adipocytes [9]. Additionally, physical activity is able to influence the production of cytokines and adipokines [10]. It has been reported that high-load training without sufficient recovery results in an imbalance between pro-inflammatory (e.g., TNFα, IL-1β, and IL-6) and anti-inflammatory cytokines (e.g., IL-4, IL-10, and IL-1) release [11,12,13]. It is plausible that the disturbance in adipocytokine balance contributes to many of the symptoms observed in OTS, emphasizing the role of AT in this condition. [14].

Studying OTS is complex because there are currently no effective biomarkers to differentiate between OR and OTS [15]. Furthermore, several biomarkers commonly used to identify OTS have been found to be modulated in both overtrained and non-overtrained individuals [16]. These features underscore the remarkable complexity of OTS, highlighting the necessity for deeper investigation into OTS markers, considering also the endocrine response of AT to training.

Although a series of contributing factors, signs/symptoms, and resulting dysregulated mechanisms of OTS have been the subject of several relatively recent reviews, the role of AT in OTS has not yet been reviewed. Moreover, diagnosing and treating OTS remains challenging for the scientific community; therefore, it could be useful to identify novel markers that can be helpful for an early diagnosis of OTS. In this context, our focus is on exploring the relationship among exercise intensity, AT activity, metabolism, and their implications in OTS. Additionally, we will delve into the role of some adipokines, such as adiponectin, leptin, resistin, growth differentiation factor 15 (GDF-15) and irisin in physical activity and as potential biomarkers for OTS.

## 2. Results

### 2.1. The Main Theories Underlying OTS

According to the latest guidelines on OTS, various hypotheses have been proposed to elucidate the nature of this syndrome [3]; however, the exact mechanisms underlying the complex pathophysiology of OTS remain unclear. Following, we will briefly describe the main theories underlying OTS.

- Glycogen Hypothesis: Insufficient muscle glycogen levels can significantly impair performance by depriving the muscles of sufficient fuel to sustain the workload. As glycogen serves as a primary energy source for muscles, its inadequate availability can hinder the ability to maintain intensity and prolong exercise duration, ultimately impacting overall performance and contributing to worsening fatigue [17,18]. Although low muscle glycogen levels may be linked to exercise-induced fatigue, the connection with OTS seems tenuous. Snyder et al. reported that even athletes with elevated carbohydrate consumption and normal glycogen levels can potentially develop OTS [18]. Furthermore, long-term carbohydrate supplementation in rats as an intervention to prevent or mitigate OTS attenuated OTS-induced performance decrements but did not reach statistical significance and was not able to protect against muscle damage [19].
- Central Fatigue Theory: Fatigue is a complex phenomenon, with several interactions among central and peripheral factors. In the presence of prolonged and excessive training, concurrent with other stressors and insufficient recovery, performance decrements can result in chronic maladaptation that can lead to OTS [20]. OTS typically involves disturbances in mood, sleep, and behavior [2,21,22]. The main neurotransmitter implicated in the regulation of these functions is serotonin (5-HT) [23]. An increase in the synthesis of 5-hydroxytryptamine (5-HT) in the central nervous system (CNS) can give rise to OTS. Physical exercise diminishes the levels of BCAA by increasing their oxidation into glucose; this favors the entry of tryptophan into the brain, followed by its conversion into 5-HT [24]. Increased 5-HT synthesis in the brain has been positively correlated with fatigue [25,26]. Literature data showed how administering serotonin reuptake inhibitors to athletes increases 5-HT levels in the brain and diminishes performance [23]. In contrast, in marathon runners, supplementation with BCAA is related to improved physical and mental health; this is potentially attributed to a reduction in 5-HT synthesis [25].
- Glutamine Hypothesis: There is substantial evidence demonstrating reductions in blood levels of the amino acid glutamine in OTS [27]. According to the glutamine theory, the frequently observed impaired immune response and associated increased rate of upper respiratory tract infections seen in OTS may be attributed to reduced blood glutamine levels; this is because glutamine is a primary fuel utilized by immune cells. Literature data have shown that extended periods of exercise can temporarily reduce plasma glutamine concentrations in athletes experiencing overtraining [28].
- Oxidative Stress Hypothesis: Changes in redox homeostasis have been documented in athletes suffering from OTS [29]. During exercise, a certain level of oxidative stress is considered beneficial as reactive oxygen species (ROS) released from damaged muscles play a role in regulating cellular repair [30,31]. Additionally, exercise-induced ROS serves as a potent physiological stimulus to increase the expression of antioxidant enzymes, thereby enhancing antioxidant defense mechanisms [32]. However, excessive physical exercise can be detrimental for athletes, as it leads to a chronic and potentially dangerous elevation of ROS levels [33]. Athletes undergoing OTS seem to be more vulnerable to oxidative damage due to diminished responses to exercise-induced stress [33]. This increased vulnerability may result in oxidative stress becoming pathological, leading to inflammation and muscle fatigue, ultimately inhibiting athletic performance [33]. The relationship between the increased oxidative stress state and OTS remains still unclear, and there is limited clinically relevant research to elucidate whether it acts as a trigger or a consequence of OTS.
- Autonomic Nervous System Hypothesis: An imbalance in the autonomic nervous system could explain some symptoms of OTS. Specifically, a reduction in sympathetic activation and an increase in parasympathetic dominance may contribute to performance inhibition, fatigue, and depression; reduced nocturnal urinary excretion of catecholamines is also observed [4]. Heart rate variability (HRV) is utilized as an indicator of autonomic function [4]. Studies employing HRV have suggested that the impact of intense training on automatic control can be reversible. Restoring a balance between sympathetic and parasympathetic forces may be attainable with a week of rest [7].
- Hypothalamic Hypothesis: The hypothalamic hypothesis suggests that disruptions in the hypothalamic-pituitary-adrenal (HPA) and hypothalamic-pituitary-gonadal axes may contribute to OTS. Endurance athletes often exhibit changes in the function of the HPA axis, and overtrained athletes may experience variations in several hormones, such as cortisol, adrenocorticotropic hormone, and testosterone. However, the existing data about these hormonal changes are contradictory, and alterations in these axes are influenced by many factors, such as exercise capacity and other hormonal levels [34].
- Muscle Damage Hypothesis: It is widely known that exhaustive exercise is linked with increased oxygen consumption in skeletal muscles, elevated lipid peroxidation, and inhibition of key mitochondrial enzymes [35]. Endurance training, when not properly balanced with adequate rest, typically does not lead to functional damage and promotes muscular maladaptation along with oxidative stress generation and a reduction in the muscle defense system [36]. Under normal training conditions, muscle fibers regenerate through the activation of satellite cells, resulting in the generation of new differentiated myofibers [37]. Conversely, continued intense training combined with inadequate rest can lead to a decrease in satellite cell numbers [38], reducing the formation of new fibers and the appropriate regeneration of damaged ones, thus contributing to the development of OTS.
- Cytokine Hypothesis: The cytokine hypothesis states that the process of muscle contraction and repetitive joint action leads to tissue microtraumas, initiating physiological adaptation via the activation of local inflammatory response and recruitment of cytokines and interleukins such as IL-1 beta, IL-6, and TNF-α [39]. Continued rigorous training coupled with insufficient rest can exacerbate this inflammatory response, leading to a chronic and pathological condition [40]. This theory has been extensively supported by several literature studies. High levels of pro-inflammatory cytokines such as IL-1 beta, IL-6, and TNF-α are also linked to an increased restriction of food intake and alteration of cellular catabolism, leading to a reduction in glycogen stores [41]. In addition, some cytokines induce a reduction of GLUT-4 transporters in stressed muscles, interfering with glucose transport for glycogen synthesis. This process may contribute to excessive muscular fatigue in overtrained athletes [42].

Furthermore, in overtrained athletes elevated levels of pro-inflammatory cytokines seem to be related also to low availability of glutamine, a precursor for the synthesis of several inflammatory proteins up-regulated in overtraining [43].

Finally, the inflammatory role of cytokines may also be linked to behavioral and psychological changes seen in OTS. Pro-inflammatory IL-1 beta and TNF-α act on the brain, leading to reduced appetite, sleep disturbance, and depressed mood [44].

In summary, elevated circulating pro-inflammatory cytokine levels coordinate the whole-body response to excessive training by (a) inducing some alterations, affecting food restriction and cellular catabolism; (b) interacting with the CNS and promoting a set of behavioral and psychological changes that sustain systemic inflammation; and (c) influencing the immune response.

Table 1 outlines the alterations in key parameters associated with the main theories of OTS in relation to physical exercise, training, and overtraining.

### 2.2. Uncovering OTS Markers: Biochemical, Immunological, and Hormonal Responses

Changes in biochemical, immunological, and hormonal responses have been observed in individuals with OTS and have been investigated as potential biomarkers for its identification [34].

- Biochemical markers, including creatine kinase (CK), urea, uric acid, ammonia, blood lactate, and plasma glutamine, have undergone extensive study across various athletes [57,58]. These markers are already used in many areas of sport to evaluate an athlete’s training response, helping coaches to identify workload and prevent injuries [59]. CK activity reflects both the intensity and volume of exercise. It is important to note that some athletes may show minimal increases in CK activity, making elevated CK activity alone insufficient to conclusively indicate OTS [60]. With regards to urea, increased concentrations may occur in response to elevated training loads, particularly in intensive endurance training, due to enhanced protein catabolism and gluconeogenesis [61]. Ammonia levels decrease at rest in athletes with OTS, although this finding is inconsistent, while uric acid levels do not show significant changes in overtrained athletes [62]. Marked responses were also observed in muscle damage markers such as blood lactate [63]. Lactate is typically released after exhaustive exercises and in smaller amounts during mild to moderate activities. Its levels are exponentially proportional to the level of activity. While several studies have reported reduced lactate levels in OTS along with impaired anaerobic lactate performance, Cadegiani et al. found increased lactate levels in OTS, likely due to impaired muscle recovery [34]. However, analyzing lactate levels post-exercise precludes the examination of lactate clearance and dynamic metabolism during rest periods. Nevertheless, numerous other metabolic variables also influence blood lactate, making it ineffective in identifying OTS on its own. Regarding glutamine levels, it has been reported that overtrained athletes exhibit low levels of plasma glutamine [6]. Changes in glutamine can also occur after physical trauma, inflammation, and infection [6]. Furthermore, plasma glutamine is also influenced by diet; in fact, it temporarily increases after the consumption of protein-rich foods [6]. Thus, the debate remains ongoing regarding whether glutamine can be reliably used as a marker of impending overtraining.
- Oxygen free radicals generated during exercise contribute to muscular fatigue and are implicated in muscle damage [30]. Overtrained athletes presented higher indices of lipoperoxidation, such as thiobarbituric acid reactive substances (TBARS) and the blood ratio of reduced vs. oxidized glutathione (GSH/GSSG ratio) [57]. Literature data reported that continuous submaximal training led to a decrease in GSH and an increase in GSSG, followed by a decrease in the GSH:GSSG ratio [64]. Generally, athletes who experienced OTS exhibit higher exercise-induced variations also in TBARS levels compared to well-trained ones [4]. Further studies are needed to determine if these indices could serve as valuable markers of OTS.
- Immunological markers are highly responsive to various forms of stress, including both physiological and psychological stressors. Athletes experiencing OTS are frequently reported to be immunosuppressed. As a result, immune parameters could potentially serve as indicators of stress levels in the context of exercise training. Recent investigations have focused on salivary immunoglobulin A (IgA) even if findings regarding salivary IgA levels in OTS athletes have been inconsistent: one study documented a significant reduction in salivary IgA levels among athletes experiencing overtraining symptoms [63], Halson SL et al. did not observe a statistically significant decrease following intensified training in a group of cyclists [65]. On the other hand, exercise and training induce changes in circulating numbers of lymphocyte subsets [66]. With heavy training, the T-lymphocyte CD4+/CD8+ ratio typically decreases [67]. However, this ratio seems to be similar between athletes diagnosed with OTS and well-trained athletes [68]. Literature data revealed that the expression of certain proteins on the surface of T-lymphocytes may be more sensitive to differentiate between the OT athletes and healthy ones [69]. Specifically, the expression of CD45RO+ on CD4+ cells was significantly higher in athletes experiencing OTS compared to healthy and well-trained controls [70]. Using this indicator, OTS could be identified with high specificity; however, further in-depth studies are needed.
- Hormonal markers are often considered potential tools for identifying OTS, yet they are susceptible to numerous confounding variables, such as diurnal and seasonal timing. Among the hormones most investigated in OTS are cortisol, adrenocorticotropic hormone (ACTH), and human growth hormone (HGH) [34]. Regarding serum cortisol levels concerning OTS, literature data are inconsistent. While some studies report no change, others indicate increases, decreases, or variable responses. Evidence also suggests ACTH and GH deficiency in athletes with OTS. Specifically, ACTH and GH responses to insulin-induced were found to be lower in overtrained athletes compared to healthy, well-trained controls [56]. Unfortunately, current data are inconsistent in some cases, as they depend on various factors, including individual training capacity, intrinsic vulnerability to stressful factors, and other hormonal levels [71].

In many studies, athletes with OTS have also shown a reduction in nocturnal catecholamine excretion, suggesting a decrease in intrinsic sympathetic activity [56]. Conversely, a few data have indicated elevated plasma norepinephrine levels [72]. In summary, basing the OTS identification on the levels of resting blood hormones is not a reliable approach. Given the considerable variability, standardization of reference ranges for most of the mentioned markers is challenging, complicating their use as reliable indicators of impending OT. Consequently, much attention has been directed towards developing novel biomarkers as possible indicators of excessive training stress with limited success to date. 

It has been established that physical activity exerts its effects also through the regulation of endocrine function of adipose tissue, making adipokines an intriguing molecular target. Indeed, adipokines may reflect physiological alterations such as muscle damage or inflammatory responses that commonly follow strenuous exercise [73]. 

The discovery of new biomarkers holds promise for enhancing the assessment of training load, recovery, and overall health status. Therefore, investigating adipokines as potential markers in athletes undergoing intensified training regimens may enable sports scientists to screen athletes more effectively for the onset of OTS. The responses of adipokines to exercise, both under normal and excessive training loads, are outlined below.

### 2.3. Adipose Tissue in Training, Reaching, and Overtraining 

Adipose tissue (AT) is a multifunctional dynamic organ, metabolically active, that can respond to changes in energy demand [74]. It is involved in numerous physiological and pathologic processes related to the regulation of energy metabolism, insulin sensitivity, inflammatory response, and thermogenesis [74]. Classically, it is divided into white adipose tissue (WAT) and brown adipose tissue (BAT) [75]. In particular, WAT is composed of unilocular adipocytes, depots of stored energy in the form of triglycerides [76]. On the contrary, BAT is composed of multi-locular and mitochondrial-rich adipocytes involved in thermogenesis and energy expenditure [77]. Both tissues, WAT and BAT, are defined as metabolically active organs because both secrete cytokines, adipokines, and batokines, respectively, to maintain nutritional homeostasis and to regulate multiple-organ crosslinking in pathophysiological conditions [78,79]. The classical view of brown and white fat cells when a third type of fat cells termed ‘brite’ (brown-in-white) adipocytes was described [80]. These cells share biochemical features and the thermogenic potential with brown adipocytes but are derived from different precursor cells [81]. The possibility to differentiate adipose stem cells into brite adipocytes (browning of white fat) and to induce thermogenic activation and augment energy expenditure is currently considered an important and promising approach to combat obesity.

Numerous adipokines have been identified to date, participating in several metabolic and inflammatory processes, as well as contributing to the normal homeostasis of various organs and tissues [82]. Some adipokines, i.e., leptin, resistin, visfatin, chemerin, and adipsin, exhibit mainly pro-inflammatory effects, while others, such as adiponectin and IL-10, primarily act as anti-inflammatory factors [82]. AT adipokines act both locally and distally with autocrine, paracrine, and endocrine effects [83]. 

Batokines spurred a new wave of interest in their effects on modulating metabolism, such as fibroblast growth factor 21 (FGF21), bone morphogenetic protein 8B (BMP8b), interleukin-6 (IL-6), vascular endothelial growth factor A (VEGFA), insulin-like growth factor 1 (IGF-1), neuroregulatory protein 4 (NRG4), the lipokine 12,13-diHOME [84]. 

The different functions of AT are also involved in response to physical activity. In fact, it is well-established that physical activity can positively remodel AT. Several molecular mechanisms are responsible for this AT remodeling: 1. activation of lipolysis and reduction of adipocyte size and/or number [85]; 2. modulation of the expression of thermogenic genes in WAT with consequent increase of the body’s energy expenditure and energy expenditure [86]. The significant markers expressed during AT remodeling are uncoupling protein 1 (UCP1), a key player of thermogenesis [87]; peroxisome proliferator-activated receptor gamma (PPARγ), which regulates glucose and lipid metabolism [88]; and its coactivator 1 α (PGC1α), which drives the thermogenesis development [89]. Moreover, the increase in irisin caused by exercise induces the browning process, and fibroblast growth factor 21 (FGF21) plays a significant physiological role in WAT thermogenesis [90]. 

### 2.4. Regulation of the Secretion of Adipokines and Batokines

An increase in the secretion of anti-inflammatory adipokines such as adiponectin and a decrease in pro-inflammatory ones such as leptin and visfatin have been suggested. Acute exhaustive and endurance exercises induce a pro-inflammatory response in AT [91]. On the contrary, correct training induces an anti-inflammatory response in the AT [92]. As said above, prolonged periods of severe training and short recovery time can lead athletes to OTS, which is characterized by declining performance and is accompanied by biochemical modulations associated with elevated levels of circulating cytokines [93]. 

Numerous studies are investigating the possibility that exercise might also stimulate BAT to release batokines [94,95,96,97,98]. For example, acute exercise enhanced the circulating lipokine 12,13-diHOME in humans, which increased skeletal muscle fatty acid uptake and oxidation [84]. One of the most well-investigated batokines is FGF21. The predominant source of FGF21 is the liver, but FGF21 is also highly expressed in WAT and BAT [99,100]. Although acute and chronic exercise increases serum FGF21 in humans [101,102], the liver has been indicated as the primary source of FGF21 in response to exercise, while the effects of exercise in the release of FGF21 from BAT are still unclear. Similarly, for VEGFA, another batokine, how exercise regulates its levels is still unknown [103].

The above-mentioned adaptations of AT in response to exercise determine athletic performance [104]. Here, we deal with the impact of WAT production of adipokines such as adiponectin, leptin, resistin, growth differentiation factor 15 (GDF-15), and irisin on exercise performance and the inter-individual variability to the exercise-mediated stress response.

### 2.5. Adiponectin in Physical Activity and in OTS

Adiponectin is the most abundantly produced adipokine. Firstly, recognize to have significant metabolic actions (increase of insulin sensitivity), successively adiponectin has been found to possess important anti-inflammatory properties [105,106]. 

It is largely known that adiponectin is strictly related to body weight and body composition since adiponectin serum concentrations are inversely related to BMI and body fat [107]. Adiponectin exists in 3 major oligomer forms: a low–molecular-weight, a medium–molecular-weight, and a high–molecular-weight form, which has been suggested to be the most active and correlated with variables related to glucose and lipid metabolism [108]. Besides the relation between adiponectin and body composition, it has been hypothesized that physical exercise exerts its beneficial effects through the regulation of adiponectin, making this adipokine an interesting molecular target. Indeed, when considering physical activity as a non-pharmacological intervention for the management of metabolic disorders, metabolic improvements mediated by physical activity are associated with changes in adiponectin serum levels [109,110,111,112].

Regarding athletes, the usefulness of adiponectin evaluation in relation to performance level is still a matter of debate. In general, peripheral signals (hormones and cytokines) may be used to reveal the condition of the athlete as the result of several months of prolonged exercise training [113]. Therefore, the possibility of using these peripheral signals as markers of training stress (and possible overreaching/overtraining) in elite athletes is under consideration. 

Although adiponectin concentration has been shown to increase acutely as a result of resistance exercise among weight-trained athletes [114], studies comparing the effects of endurance or resistance training on resting adiponectin concentration have found little effect over 12 and 16 weeks suggesting that longer periods are necessary to produce a modulatory effect in adiponectin levels [115].

Nevertheless, it appears to be important considering adiponectin levels at both baseline and post-exercise. At baseline, a slight increase in adiponectin levels has been reported with training stress; the slight increase might be explained by the fact that well-trained athletes present relatively high baseline adiponectin concentrations compared with the baseline adiponectin levels in healthy but less-trained subjects [116,117,118,119]. Such an increased concentration of adiponectin would be a potentially advantageous trend because adiponectin is generally associated with reduced inflammation [120,121]; on the other hand, it could be related to increased joint inflammation, which is a more negative outcome of training.

Taking into account adiponectin regulation post-exercise, higher-performance athletes demonstrated higher exercise-induced adiponectin concentrations, while lowered adiponectin post-exercise was found in lower-performance athletes as a sign of inadequate recovery and inadequate performance level of these athletes [122].

These findings suggest that training could modify adiponectin response depending on the performance level of athletes and that decreases in postexercise adiponectin may be a sign of inadequate recovery. However, further studies are needed before any conclusions may be made.

How different types of sports might impact adiponectin regulation is also still under investigation. Kraemer et al. [123] demonstrated that well-trained runners completed strenuous running at 60% to 100% of VO2 max, elicited a small (10%) but significant increase in postexercise adiponectin. Similarly, Jamurtas et al. [124] have demonstrated that a mean of approximately 15% increase in adiponectin concentration occurred after the first 30 min of recovery of on-water sculling at 75% VO2 max for approximately 30 min. Similarly, another study also demonstrated a delayed increase in adiponectin after the first 30-min recovery of maximal 6000-m ergometer rowing (∼20 min) [125]. Interestingly, adiponectin has been shown to have an inverse relationship with resting cortisol concentrations [126]. Fallo et al. demonstrated that glucocorticoids inhibit adiponectin due to both exogenous administration to healthy subjects and endogenous cortisol hyperproduction [126]. Prolonged sustained fatigue, often associated with increased glucocorticoid production and with an increase in an athlete’s overtraining potential, can be accompanied by increased psychological stress. Increased cortisol and low adiponectin concentrations may be associated with psychological manifestations accompanying accumulated training-induced fatigue. For example, lower adiponectin concentrations have been associated with increased susceptibility to depressive behaviors and impaired glucocorticoid-mediated negative feedback on the hypothalamic–pituitary axis [127], which is a process related to cumulative fatigue. Therefore, decreased cortisol and corresponding increased adiponectin concentrations may be indicative of lower psychological stress levels, but this is not yet well studied in athletic populations. 

**Figure 2:**
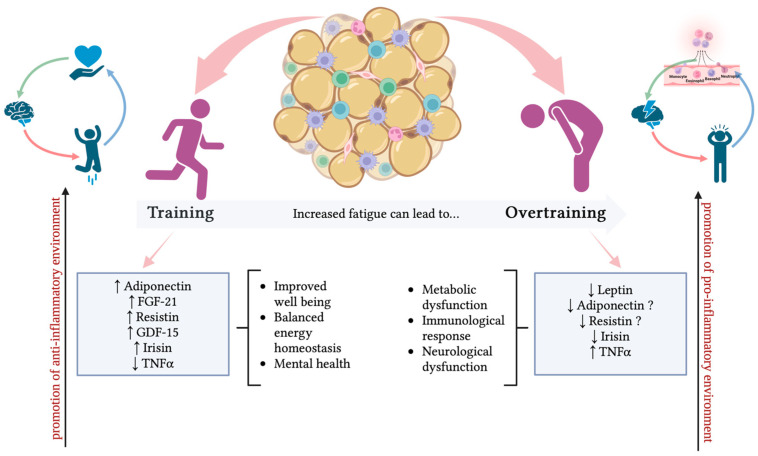
Adiponectin, FGF-21, resistin, GDF-15, irisin, and TNF-α are modulated by training and OT. This schematic representation illustrates the altered levels of the aforementioned adipokines, along with their effects on general well-being. The upward arrow (↑) indicates elevated levels of each adipokine; the downward arrow (↓) indicates reduced levels of each adipokine; the question mark (?) indicates a lack of relevant literature data supporting an increase or decrease in each adipokine. The figure was created using BioRender.com.

In short, well-trained athletes have relatively high baseline adiponectin concentrations. Therefore, basal adiponectin seems to remain stable in conditions of high training stress. Accordingly, basal adiponectin is not a good marker of energy homeostasis in athletes, while post-exercise is a reasonable field of research. Figure 2 summarizes the regulation of adiponectin in response to training and OT and its related effects on health.

### 2.6. Leptin in Physical Activity and OTS 

The adipocytes secrete leptin into the bloodstream proportionally to fat mass; hence, leptin levels are strongly correlated with adiposity [113]. Functionally, leptin regulates the balance between food intake and energy expenditure; besides metabolic functions, leptin regulates neuroendocrine function and oxidative stress [128]. Among sedentary populations, leptin has been positively correlated with a wide range of adverse health outcomes [129]. Although a positive relationship between adiposity and leptin concentration in sedentary people has been clearly described, but not always in athletes [130,131]. Among track athletes, contrasting results have been reported. At basal levels, leptin concentrations in athletes appear to be relatively low compared with sedentary individuals [129,130,131]. Peresghin et al. found elevated concentrations of leptin [132] resulting from a non-obesity-linked inflammation (e.g., perhaps training-related). Two studies examining leptin response to resistance training found no significant changes, while a study by Guadalupe-Grau et al. found significant changes in serum leptin levels in females after 9 weeks of resistance training (no significant differences were found in the males) [133]. Varady et al. investigated acute adipokine responses in males placed in different exercise groups (resistance exercise, resistance exercise plus running, running only) [134]. The 2 groups that involved resistance training demonstrated statistically significant increases in adiponectin, while leptin did not change. 

A negative correlation between leptin and performance [113] and a dose-response relationship of leptin with weekly training volume [135] have been described. Mechanistically, the decrease in leptin levels post-exercise seems to be strictly related to the reduction of energy availability [113]. Regarding overtraining, Joro et al. reported that in overtrained athletes compared to healthy controls, leptin levels are lower at rest, and pro-inflammatory cytokine response to acute exercise may reflect a chronic maladaptation state in overtrained athletes [136]. In contrast, the accentuation of IL-6 and TNF-α responses to acute exercise seemed to be associated with the progression of recovery from overtraining [113]. In addition, Yamaner reported that the intense wrestling training during the camping period brought about weight loss and decreased leptin levels. The authors reported that during the preparation camp period, intensive exercise programs caused rapid weight loss, and it was determined that serum leptin levels decreased significantly in wrestling women [137]. 

In summary, it appears that basal leptin is lowered in endurance athletes and post-exercise when energy expenditure is reduced, depending on exercise intensity, and might be related to limited energy availability. This could be indicative of an inadequate recovery in overtrained athletes. Whether training stress and decreased energy availability in athletes can be monitored through leptin levels needs to be further investigated and confirmed. Figure 2 summarizes the regulation of leptin in response to training and OT and its related effects on health.

### 2.7. Other Adipokines in Physical Activity and in OTS

- TNF-α is a 26-kDa transmembrane multi-functional cytokine that, after a proteolytic cleavage, produces a 17-KDa soluble molecule. Biological functions include the regulation of many cellular and biological processes, such as immune function, cell differentiation, proliferation, apoptosis, and energy metabolism. Beyond macrophages as a well-known source of TNF-α, mature adipocytes and stromovascular fraction (SVF), which contains preadipocytes, endothelial cells, smooth muscle cells, fibroblasts, leukocytes, and macrophages in AT, also represent an important source of TNF-α production [138,139]. Generally, catabolic states are associated with lower levels of soluble circulating TNF-α compared to the obese state characterized by higher levels [140]. Secreted TNF-α then acts both on AT and peripheral tissues through the binding with two receptors, TNFR1 and 2 [138]. How exercise can modify the expression of AT-derived TNF-α is controversial. Regarding circulating levels, the response of TNFα to exercise largely depends on the intensity of training. Regular moderate exercise causes TNFα suppression through IL-6 [140,141], while results are different for acute exercise, such as marathon running, where TNF-α concentrations strongly increase [142]. Interestingly, the increase in TNF-α levels is higher with strenuous training caused by muscle damage during exercise [143]. Thus, in OTS, TNF-α is considered a key cytokine included in the “cytokine hypothesis” of OTS, and thus, these proinflammatory cytokines are significantly associated with measures of depressed mood, sleep disturbances, and stress in OTS athletes [144]. As said above, the cytokine hypothesis considers that an imbalance involving excessive exercise and inadequate recovery induces musculoskeletal trauma, increasing the production and release of proinflammatory cytokines, mainly IL-6, TNF-α, and IL-1beta, which interact with different organic systems, initiating most of the signs and symptoms linked to performance decrement [144].

The specific modulation of TNF-α produced by adipose tissue during exercise was examined by Nara et al. [145] using an animal model of insulin resistance that was produced by feeding rats a diet high in sucrose. The authors showed that 12-week voluntary running exercise significantly increased both TNF-α protein and mRNA in the mesenteric fat of insulin-resistant rats compared to non-exercised fed rats; the authors suggest that the up-regulation of TNF-α in mesenteric fat may be a compensatory mechanism for the reduction of fatty acid in AT and this change could control metabolic homeostasis during exercise to modulate a hyperinsulinemic state [145]. The precise cellular source of TNF-α from AT, however, is still unknown. With specific regard to OTS, to our knowledge, no studies have analyzed the contribution of AT to TNF-α increase.

- Resistin is a cysteine-rich hormone secreted from white adipocytes and macrophages in humans. Resistin forms multimeric structures (hexamer and trimer) in circulation [146]. Resistin is involved in insulin resistance, but it is also involved in inflammatory responses in humans [147]. High resistin levels induce insulin resistance and exert proinflammatory effects [148].

Prestes et al. evaluated the effects of resistance training on cytokines, leptin, and resistin in women (not athletes) finding that resistin and leptin decreased 48 h and 24 h after compared with baseline, respectively [149]. There was a decrease also in resistin levels after 24 and 48h compared with baseline suggesting a reduction in pro-inflammatory cytokines by the innate immune system. However, the study was not conducted on athletes. In marathon athletes, resistin was evaluated in four studies showing increased concentration after running [150,151,152,153]. Moreover, clinical studies have shown that the increase in resistin after exercise is associated with a reduction of fatigue and a reduction of musculoskeletal and joint inflammatory events [154]. These data encourage the evaluation of resistin post-exercise in athletes although clear data on OTS are not available.

- Growth differentiation factor 15 (GDF-15) was first recognized in 1997 as a macrophage inhibitory cytokine-1 expressed in multiple tissues, including skeletal muscles [155]. Nowadays, it is known that it is also produced by adipose tissue under stress conditions to maintain cell and tissue homeostasis [156]. Recent studies also suggested that GDF-15 functions as an exerkine secreted by AT, exhibiting a possible protective role in exercise-induced muscle injury or inflammation. Normally, circulating blood levels of GDF-15 are quite low but it can be measured before and after exercise to assess the exercise-induced stress response intensity of each individual. In detail, high GDF-15 levels have been associated with lower muscle mass and strength, frailty, and declining physical function [157,158,159,160]. Tchou et al. found a strong increase in plasma GDF15 at the end of an ultramarathon foot race in male athletes [161]. In professional rugby players, circulating GDF15 was significantly increased after a session of intense training [162], and an increase in serum GDF15 was also observed in football players of the Spanish Football League 12 h after a match [163]. Recently, plasma GDF15 concentrations were found to gradually increase during and after a controlled vigorous submaximal exercise in young, healthy males [164].

On the other hand, in animal models, cachectic mice treated with the anti-GDF15 antibody exhibit body weight gain with near-complete restoration of muscle mass and markedly improved muscle function and physical performance [165].

Although it has been proposed that GDF-15 levels are related to inflammatory states, recently, it has been found that its relation to performance is independent of inflammation [166]. Since GDF-15 is expressed in most organs, including the bladder, kidney, colon, stomach, liver, gall bladder, pancreas, endometrium, and muscles, and cell types, including cardiomyocytes, adipocytes, macrophages, endothelial, myocytes, and vascular smooth muscle cells, it is quite difficult to establish which organ contributes to a greater extent. However, Gil et al. proposed that the substantial contribution to elevated levels of GDF-15 post-exercise is not muscle, highlighting the possibility that adipose tissue could be implicated [163]. Importantly, Jena et al. found GDF-15 implicated in the thermogenic activity of BAT and the browning process [167], suggesting that it impacts exercise performance acting at multiple levels, i.e., muscle damage and AT browning.

- Irisin is a cytokine produced by both muscle and AT during exercise [168]. Irisin is considered a thermogenic adipomyokine, able to improve glucose and lipid metabolism and ameliorate the effects of obesity-driven inflammation [169]. Irisin levels can be induced by exercise, both acute and resistance [170]. Irisin expression is regulated by the transcriptional coactivator peroxisome proliferator-activated receptor-gamma coactivator 1 alpha (PGC1-α) [171]. An increase in PGC1-α expression in the muscle of transgenic mice is associated with WAT browning in a process mediated by irisin [170]. Additionally, PGC1-α is associated with exercise-mediated benefits and energy metabolism, leading to the hypothesis that exercise may stimulate irisin release [170].
  Elite athletes showed higher levels compared to sedentary and recreational subjects, suggesting a correlation between sports performance, insulin sensitivity, and irisin levels [172,173].

Functionally, exercise-induced irisin activates anti-inflammatory pathways and may play an essential role in improving the outcomes of inflammatory conditions [174]. In addition to the reduction of the inflammatory state, irisin is considered an insulin-sensitizing molecule and a favorable factor for cognitive health [175]. Therefore, irisin is a key hormone induced by exercise, clearly contributing to mediating its positive effects. However, it is noticed that the production of serum irisin is due to both muscle and AT and differentiating between the two sources is unreasonable; also, the amount of irisin produced by AT has been estimated at very low levels [176]. Regarding OTS or reduction of performance, to our knowledge, only one study investigated irisin levels, finding a downregulation of its serum levels in OTS concerning healthy athletes with a specific correlation with oxidative stress markers [177]. In conclusion, the dosage of irisin is quite useful both in metabolic patients and in athletes; in the latter, it could help to discriminate between OTS and healthy athletes and to highlight an oxidative stress status.

Figure 2 summarizes the regulation of the aforementioned adipokines in response to training and OT, along with their related effects on health.

## 3. Discussion

High levels of training volume and overall workload are generally effective in increasing performance in athletes. However, unsuitable training protocols could lead to OTS syndrome. The involvement of AT through alteration of adipokines secretion in OTS remains to be further clarified; certainly, during OTS there is a dysregulation in cytokines and immune response in which AT also participates. In particular, adiponectin and leptin are modulated by weight loss that occurs as a result of overtraining, and also these adipokines are modulated following a hypothalamic response. Given the complex etiology of OTS, the role of AT in OTS has not been previously examined. In this review, we examined the differential expression of adipokines during different modalities of physical activity and, thus, potential biomarkers of good health or OT. The complex network of adipokines and the mutual interactions between adipokines in coordinating inter-organ crosstalk represent another important area to be explored further. In fact, the improvement of insulin sensitivity, glycemic control and oxidative stress can be monitored by different metabolites or bio-factors, such as those produced and secreted by AT. Overall, these molecules can potentially contribute to the development of different solutions for both diagnosis and/or therapeutic strategies of OTS. Furthermore, the demonstration that basal expression of adipocytokines is closely related to individual responses to physical exercise can potentially lead to the implementation of new personalized training protocols.

The data emerging from our review represent an interesting attempt to provide valuable information on the effects of exercise on AT endocrine production, but they also bring many challenges that represent a limitation of this study. Indeed, the expression of the different adipokines is influenced by the exercise modality, intensity, duration and by a series of still unknown factors and linked to individual features such as the sexual difference. Further investigation of these exercise variables may help to better understand and find a way for personalized exercise intervention. There is a lack of comparison regarding the effects of different exercise modalities and intensities on exerkine production. Additional limitations are the female athletes are underestimated in OTS studies and the small sample size of many studies performed on OTS athletes.

The physiological function of the majority of exerkines remains largely unexplored in the context of exercise. Future integrated studies should be exploited to dissect the complex, dynamic exerkine regulatory network functionally involved in the metabolic adaptation to exercise.

The prevalence of OTS is highest in endurance sports requiring high-volume training, such as swimming, triathlon, cycling, and rowing [8]. It is reported that 64% of female and 66% of male elite distance runners experience staleness during their competitive careers [178]. Considering this data, it is clear that the identification of novel biomarkers for OTS is an urgent and important field of research with a relevant impact on the clinical management of athletes to establish the nature of OTS and to identify an individualized signature of OTS.

The strength of the present study is that the presented data clearly define adipokines as potential biochemical markers to monitor athletes and the effectiveness and quality of the training process. The best way to monitor an athlete’s training process is to consider a constellation of markers, including the AT hormonal response related to exercise and training.

## 4. Methods

In this review, we analyzed published articles from the most recent literature, providing a balanced and comprehensive overview of the most important discoveries in relation to pathogenesis and possible biomarkers and target molecules of OTS. The databases used were PubMed, Scopus, Web of Science and Google Scholar, using appropriate keywords (OTS., adipokines, adiponectin, leptin, irisin, TNF-α GDF-15). Out of approximately 250 papers (original articles, systematic and meta-analysis reviews), 153 were chosen and considered appropriate for this focused review. Other papers related to the chosen keywords were discarded, as they were considered to be outside the scope of the research. The time period chosen for the literature search was from 1988 to date. The bibliographic research has been divided into four different steps and has followed inductive reasoning. In the first step, the research was focused on papers regarding “OTS”; in the second step, the “Adipose tissue in OTS”; in the third step, the “Biomarkers of OTS”; and in the fourth step, the “adipokines”.

## Figures and Tables

**Table 1:** The main theories underlying OTS are based on key parameters related to physical exercise, training, and overtraining.

Parameters	Physical Exercise	Training	Overtraining
Glycogen	Glycogen levels were significantly reduced immediately after exercise in both untrained and trained conditions [45].	Glycogen depletion during training occurs in an intensity-dependent manner [46].	Limited literature data exist regarding the relationship between low glycogen levels and overtrained athletes. It’s worth noting that athletes with normal glycogen levels still experience OTS [18].
Central fatigue	Physical exercise decreases the levels of branched-chain amino acids (BCAA), favoring the entry of tryptophan into the brain and its conversion into 5-HT. Changes in performance in relation to fatigue need to be investigated in response to different types of exercise [23].	Training influences the mechanism of central fatigue, which may vary depending on the type of training. There is a positive correlation between increased serotonin (5-HT) synthesis in the brain and fatigue among trained athletes [47].	Excessive levels of 5-hydroxytryptamine (5-HT) in the central nervous system (CNS) can lead to OTS [doi: 10.1249/00005768-199307000-00015] [25]. Increased concentrations of this neurotransmitter in the CNS are related to mental and physical underperformance [48].
Glutamine	The response of blood glutamine varies depending on the duration of exercise. Short-term exercise leads to increased muscle release of glutamine, resulting in elevated blood concentrations. Conversely, during long-term exercises, muscle synthesis of glutamine fails to meet the body’s demand for this amino acid, leading to a decrease in its levels [49].	Prolonged periods of intense training correlate with a reduction in plasma glutamine concentration, which has been proposed as a potential contributor to exercise-induced immune suppression and susceptibility to infections among athletes [27].	The decrease in glutamine blood levels is closely associated with OTS. Prolonged periods of exercise can lead to temporary reductions in plasma glutamine concentrations among athletes experiencing OTS [28].
Oxidative Stress	ROS are produced during exercise and play a role in modulating muscle contraction levels. However, the significant increase in ROS production during intense exercise contributes to the onset of acute muscle fatigue [50].	Training can reduce systemic oxidative stress and enhance antioxidant defenses. However, the effects on oxidative stress depend on training load, specificity, and baseline fitness levels [51].	Athletes undergoing OTS seem to be more vulnerable to oxidative damage due to diminished responses to exercise-induced stress [29,33].
Autonomic Nervous System	Exercise leads to a lower resting heart rate by boosting parasympathetic activity and reducing sympathetic activity. It also reduces plasma catecholamines and sympathetic outflow [52].	Training induces significant hemodynamic and autonomic changes, which are dependent on training intensity, frequency, and duration [52].	In OTS athletes, a reduction in sympathetic activation and an increase in parasympathetic dominance may contribute to performance inhibition, fatigue and depression; reduced nocturnal urinary excretion of catecholamines are also observed [4].
Hypothalamic hormones	Aerobic, endurance or continuous exercise can cause variations in hypothalamic hormone levels, depending on the intensity and duration of the activity [52].	The hormonal adaptations differ between acute and long-term training. Specifically, short-term training tends to elevate plasma levels of glucocorticoids, whereas long-term training is associated with reduced cortisol levels [53].	Lack of relevant data.
Cytokines	Regular exercise is associated with an increase of anti-inflammatory cytokines in circulation, while acute exercise may initiate inflammatory cascades depending on intensity and duration [54].	Training induces a temporary rise in pro-inflammatory cytokines, followed by anti-inflammatory cytokine production. This is attributed to the development of chronic low-grade inflammation through regular training, which primes the immune system and diminishes the cytokine response cascade to subsequent exercise. The cytokine response is influenced by the intensity and duration of training [55].	Injuries to the muscular, skeletal, or joint systems initiate OTS. Continuing training after an acute injury without sufficient recovery can worsen the original condition and exacerbate exercise-related injuries. These injuries prompt an increase in pro-inflammatory cytokines [56].
